# Survival Against the Odds: Successful Treatment of Incomplete Traumatic Hemipelvectomy in a 29-Year-Old Woman Following a Boating Accident

**DOI:** 10.7759/cureus.79095

**Published:** 2025-02-16

**Authors:** Max Smith, Joseph R Lewis, Taryn Bolling, Scott Brick, Patrick Leach

**Affiliations:** 1 Medical School, Kansas City University, Kansas City, USA; 2 Department of Trauma and Acute Care Surgery, Lee Health, Gulf Coast Medical Center, Fort Myers, USA; 3 Department of Infectious Diseases, Lee Health, Gulf Coast Medical Center, Fort Myers, USA; 4 Department of Wound Care, Lee Health, Gulf Coast Medical Center, Fort Myers, USA; 5 Department of Orthopedic Surgery, Lee Health, Gulf Coast Medical Center, Fort Myers, USA

**Keywords:** acute care surgery and trauma, hemipelvectomy, infection prevention and control, major lower limb amputation, major trauma, massive hemorrhage, orthopedic traumatology, traumatic hemipelvectomy

## Abstract

Traumatic hemipelvectomy (THP) is a rare, life-threatening injury characterized by the dislocation of the hemipelvis, often resulting from high-energy trauma. It carries a high mortality risk due to severe vascular injuries, with survival contingent on rapid hemorrhage control and a multidisciplinary approach. Limb salvage is rarely achievable due to the extent of the injury and complications such as infection and tissue nonviability. We present a case of incomplete THP in a 29-year-old woman who sustained severe soft tissue, bony, and vascular injuries following a boating accident. On arrival, the patient was in critical condition with a massive hemorrhage, requiring immediate intervention. A multidisciplinary approach was implemented, including damage control surgery, vascular ligation, massive transfusion protocol (MTP), and selective embolization. Despite initial efforts, her left lower extremity was deemed unsalvageable, and a left hemipelvectomy was performed. Postoperative care involved managing pedicle flap congestion with sequential wound debridement and hyperbaric oxygen therapy. The patient’s recovery included multiple surgical interventions for wound care and flap viability. Over time, viable tissue demarcation allowed for more definitive closure. This case underscores the complexity of managing THP, highlighting the importance of timely, coordinated surgical efforts, aggressive hemorrhage control, and postoperative care to prevent infection and optimize functional outcomes.

## Introduction

Traumatic hemipelvectomy (THP) is a rare and life-threatening injury characterized by complete dislocation of the hemipelvis, often involving the disruption of the iliac vessels following high-energy trauma [1,2]. The true incidence of THP is challenging to determine, as many patients succumb to their injuries before reaching the hospital [3,4]. THP can manifest as either partial or complete, with partial THP defined by the lower extremity remaining attached to the trunk by soft tissue. In comparison, complete THP involves total dissociation of the lower extremity from the body [5]. Given the severity of these injuries, immediate therapeutic action is crucial for patient survival, with early amputation proposed as a life-saving intervention by achieving rapid hemorrhage control and minimizing complications [2]. Advances in prehospital resuscitative techniques have improved survival rates, but management remains complex and requires a multidisciplinary approach, including trauma surgery, orthopedics, infectious disease, and interventional radiology [6,7]. Limb salvage, though possible with modern reconstructive techniques, often results in nonfunctional extremities, and major complications such as infection, phantom limb pain, and skin flap necrosis can arise [2,8-10]. Despite these challenges, some patients achieve positive functional outcomes with prosthetics and psychological support, and case reports have highlighted successful limb salvage and replantation in specific circumstances [6,9]. We present a case of incomplete THP in a young woman following a boating accident, detailing the multidisciplinary approach to resuscitation, surgical intervention, and postoperative care.

## Case presentation

**Figure 1:**
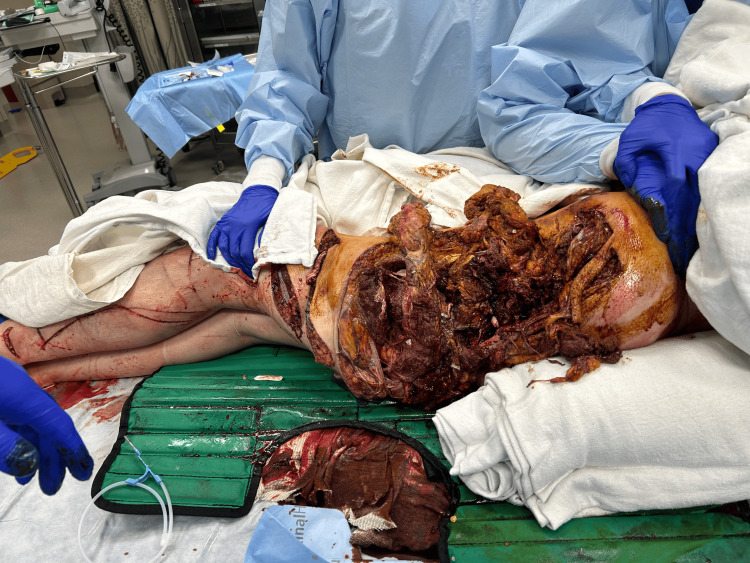
Soft tissue injury Initial presentation, with the patient in right lateral decubitus showing the upper buttocks, lower buttocks, and lower extremities.

**Figure 2:**
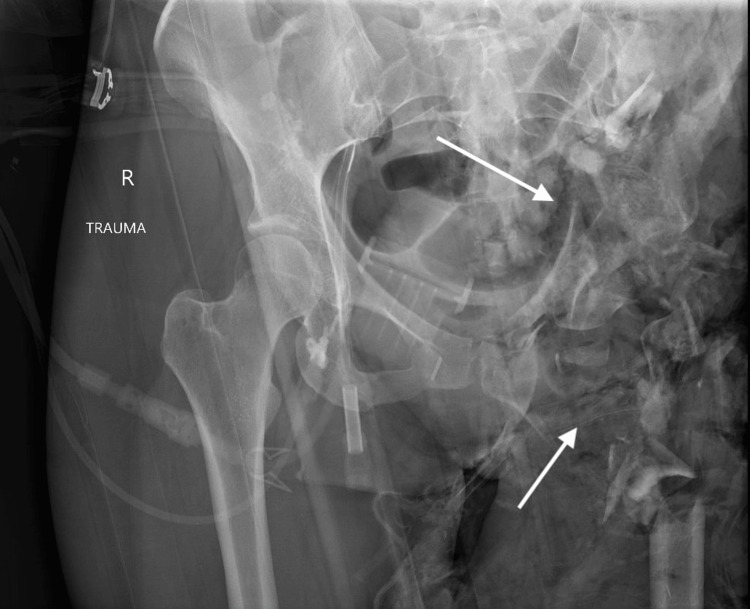
Pelvic X-ray The arrows indicate complete destruction of the hemipelvis and proximal femur.

**Figure 3:**
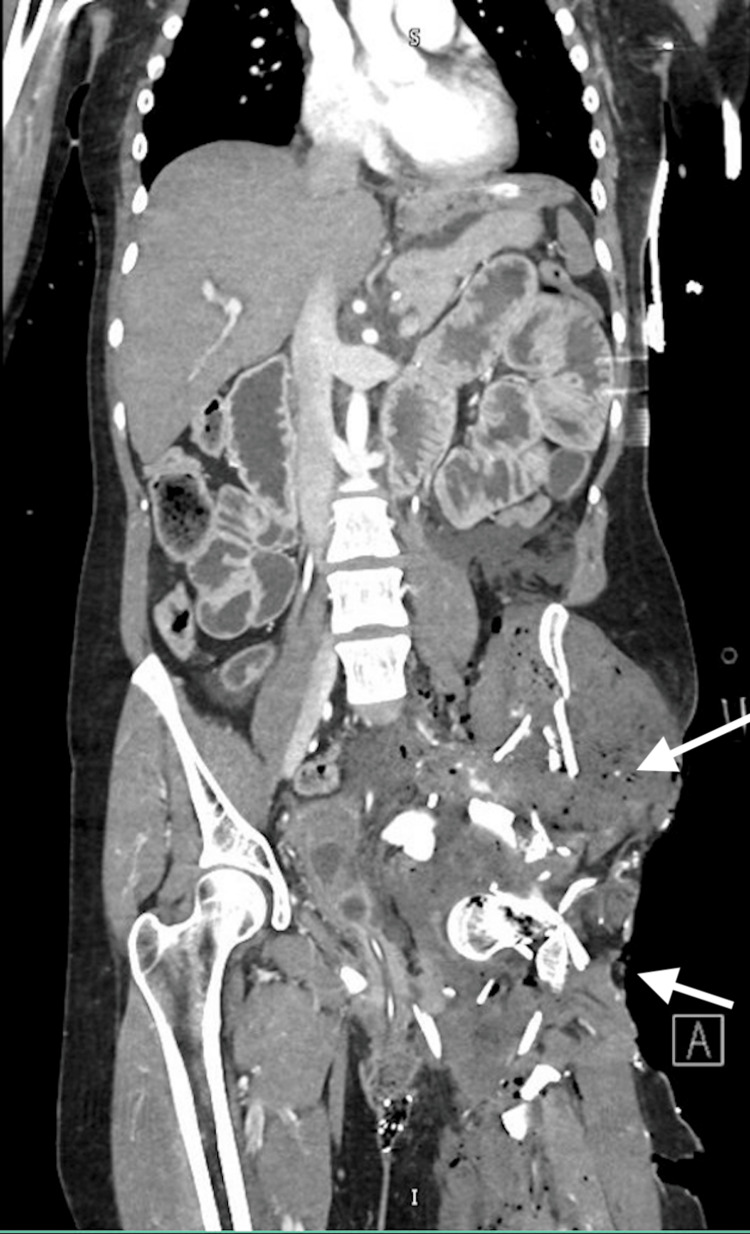
CT scan of the abdomen and pelvis with IV contrast The arrows indicate destruction of the hemipelvis and severe soft tissue injury.

A 29-year-old woman presented to the trauma bay via life flight after a boating accident, where she fell off the boat and was subsequently run over by the propeller, sustaining massive soft tissue, skeletal, and vascular injuries to the left flank, pelvis, and left lower extremity. She remained submerged in the water for an unknown period until she was rescued and transported to our trauma center with a pelvic binder in place. Upon arrival, she was hypotensive, tachycardic, and hypoxic, with a Glasgow Coma Scale (GCS) of 3 and an injury severity score (ISS) of 45. She was intubated, and the massive transfusion protocol (MTP) was activated. Ceftriaxone, vancomycin, and doxycycline were given due to extensive open soft tissue injuries and submersion in brackish waters (Figure 1). Focused assessment with sonography for trauma (FAST) was negative, and pelvic X-ray (Figure 2) in the trauma bay showed destruction of the left hemipelvis and proximal femur. The large soft tissue wound was packed with a hemostatic agent, and a pelvic binder was placed to tamponade the bleeding. She responded to MTP resuscitation and was then taken to computed tomography (CT) to assess the extent of injuries, localize bleeding, and rule out traumatic brain injury (TBI). CT revealed severe tissue trauma to the left hip, buttocks, and lower extremity with a left internal iliac laceration and pelvic hemorrhage (Figure 3) without evidence of TBI. Therapeutic intervention was discussed with orthopedic trauma surgeons, and the limb was determined to be unsalvageable. She was immediately taken to the operating room (OR) for damage control and subsequent ligation of the bleeding vessels. A midline laparotomy was performed to simultaneously evaluate for hollow viscus injury and control of bleeding. No intra-abdominal hollow viscus injury was noted. A left retroperitoneal dissection was performed to expose the left iliac and aorta. Supraceliac aortic compression was performed to control pelvic hemorrhage. Subsequently, the patient went into cardiac arrest, and CPR was started. Return of spontaneous circulation was achieved after approximately three minutes. Rapid damage control measures were taken, and ligation of the left external iliac vein and artery was performed to limit ongoing hemorrhage from the left lower extremity along with permissive hypotension. The large pelvic venous plexus was identified and ligated. No bleeding from the internal iliac artery was observable, and the area was packed to prevent ongoing hemorrhage and bowel evisceration through the hemipelvis. To provide additional tamponade, sutures in a figure-8 fashion were applied to the lower abdominal fascia. The left buttocks and hip area were inspected next, removing nonviable tissue, muscle, fascia, skin, and bone. Bleeding was identified, and it dissected into the sacral space, which was densely packed with gauze bandage rolls and laparotomy pads. The pelvic binder was then reapplied, and the patient was transferred to the trauma intensive care unit (TICU) for correction of coagulopathy as directed by the thromboelastography (TEG). Although TEG was corrected, additional bleeding was observed from the wound management device later that night, prompting the patient to be sent to interventional radiology for selective embolization of the left internal iliac artery. Final resuscitation involved transfusing just over 100 units of blood products.

**Figure 4:**
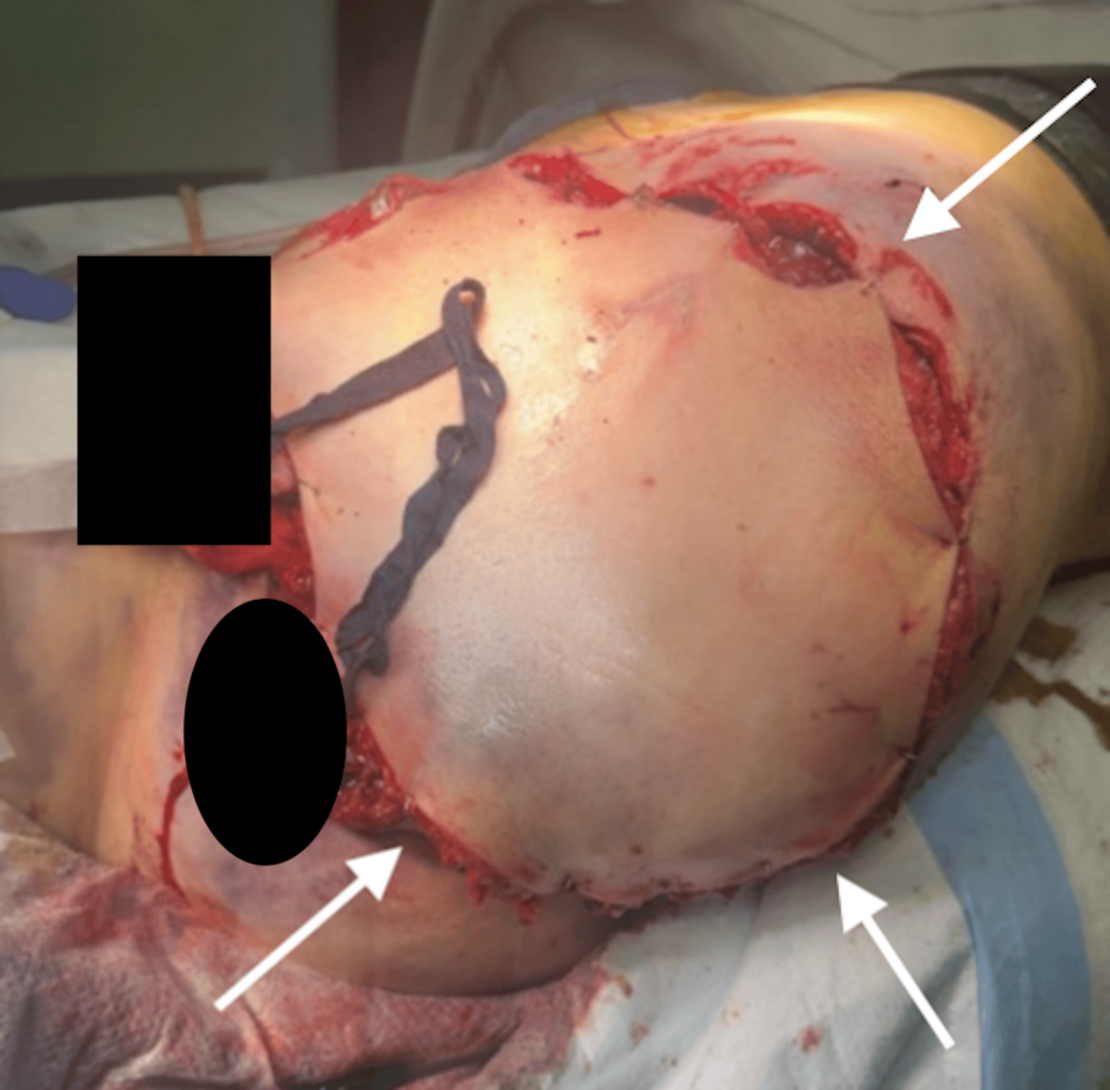
Initial post-op picture following hemipelvectomy The arrows indicate the anterior thigh pedicle loosely tacked down via staples.

On hospital day 1, the patient remained intubated, with a GCS of 11T, following motor commands and opening her eyes on her own. At this point, discussions with the orthopedic surgeon focused on the operative planning to complete the hemipelvectomy and begin soft tissue reconstruction. Creatinine phosphokinase (CPK) peaked at 15,354 less than 24 hours after the initial injury, and to prevent acute renal failure, the decision was made to take her to the OR for a left hemipelvectomy and soft tissue repair. In the OR, skin flap viability was determined via near-infrared fluorescence imaging technique on the anterior thigh, and it was decided that this would be utilized as the pedicle. The wound was explored, and devitalized tissue and bone fragments were removed from the pelvic cavity. The sartorius, adductors, gluteus maximus, and gluteus medius were dissected from what was remaining of their bony attachments to utilize as padding for the defect. Iliac vein and sacral plexus bleeding were noted, and these were ligated. At this point, the lower extremity was fully amputated. Inspection of the pelvic contents revealed a small bladder injury, longitudinal vaginal vault laceration, and minor labial lacerations that were all repaired. The mesorectum was visibly injured and contused, but this was left for the operation planned in two days. A near-full sphincter laceration was repaired with sphincteroplasty. The large defect was then heavily irrigated, packed with more hemostatic agents and laparotomy pads, and the anterior thigh pedicle was loosely tacked to the skin with staples (Figure 4). 

On hospital day 3, the patient was stable and remained intubated, with a GCS of 11T. The patient's abdomen remained open with a wound vac device in place, and plans were made to take the patient to the OR for reinspection and closure. The wound vac device was removed from the abdomen, and the abdominal contents were once again inspected. The abdominal contents were intact; the abdomen was heavily irrigated with saline and chlorhexidine solution. Due to significant rectal injury and the absence of rectal tone detected after sphincteroplasty, a loop colostomy was chosen instead of an end colostomy to facilitate potential reversal, given the extensive destruction of the hemipelvis. The abdomen was subsequently closed with a diverting colostomy. Attention was now turned to the pedicle flap, vagina, and anus. Upon gross inspection and utilizing spy fluorescence, the proximal portion of the pedicle flap appeared healthy and viable, while the distal portion showed signs of congestion. It was determined to revisit this later in the week. The vaginal vault and labia were intact and appeared more viable after the previous repair. The rectum was inspected and found to have ischemic changes and a laceration. This was subsequently debrided back to viable tissue and repaired. Plans to take the patient back to the OR and provide wound care for the pedicle and underlying contents were planned for two days from now. The patient was then moved back to the TICU and was subsequently extubated. 

**Figure 5:**
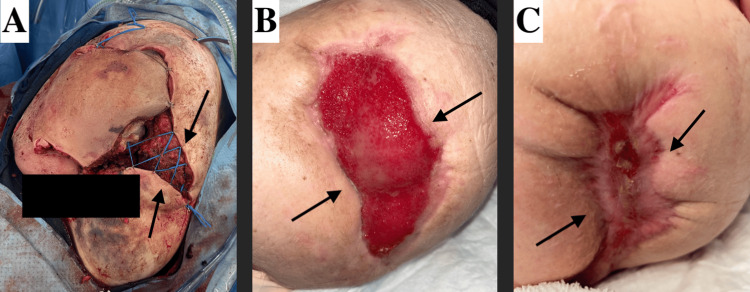
Wound healing progression (A) Arrows showing wound after pedicle demarcation, debridement, and revision on April 25, 2024. (B) Arrows showing the healing process and granulation tissue on June 24, 2024. (C) Arrows showing nearly completely healed on September 04, 2024.

Subsequent wound care operations and debridements were performed to allow for continued demarcation of viable tissue and assess the integrity of the pedicle flap. Much of the anterior pedicle remained viable over the week following the initial operation, permitting more definitive wound closure. Due to the prior demarcation of nonviable tissue, a 20 cm x 10 cm wound was left open with a synthetic bilayer wound matrix xenograft placed to facilitate coverage and support healing. Extracellular matrix (ECM) beads were also applied to promote cellular migration and tissue regeneration in the wound bed. Hyperbaric oxygen therapy was utilized to further enhance wound healing. Wound healing progression is illustrated in Figure 5. Diligent wound care was maintained, with regular lab monitoring to detect and prevent potential sepsis given the severity of the injury.

Infectious disease section 

While managing the patient’s THP, a complex infectious disease profile emerged, which was closely managed by both the infectious disease service and the pharmacy team. Initial wound cultures following pelvic incision and drainage (I&D) on April 25, 2024, revealed *Enterococcus gallinarum*, *Enterococcus hirae*, *Bacillus *spp., and *Stenotrophomonas maltophilia*. Broad-spectrum antibiotics were initiated, but subsequent cultures following pelvic I&D on April 28, 2024, showed persistent *S. maltophilia*, along with *Candida parapsilosis* and *Enterococcus gallinarum*. Further wound cultures after right leg debridement on April 30, 2024, also grew *C. parapsilosis*.

Despite repeated debridements, including another on May 03, 2024, wound cultures continued to demonstrate *S. maltophilia*, *E. gallinarum*, and *Candida glabrata*. The patient was managed with an evolving antibiotic regimen that initially included intravenous piperacillin-tazobactam, which was later transitioned to ampicillin, minocycline, and micafungin. She completed a 14-day course of antibiotics/antifungals by May 16, 2024.

Complications continued with the discovery of a left retroperitoneal abscess, necessitating aspiration and drain placement via interventional radiology on May 17, 2024. Cultures from the abdominal wound again grew *E. gallinarum*. A seven-day course of intravenous ampicillin was completed on May 23, 2024, and she received a five-day course of ciprofloxacin and metronidazole for additional gram-negative and anaerobic coverage, finishing on May 26, 2024.

Interestingly, despite the patient’s exposure to warm waters in Florida, no *Vibrio* or *non-tuberculous mycobacteria* (NTM) were identified in her wound cultures. Given the geographical risk factors and the nature of the injury, this absence was somewhat unexpected, as these organisms are known to be present in such environments and often complicate water-related trauma cases. This highlights the variability in microbial colonization and the importance of targeted antimicrobial therapy based on culture results that were obtained.

## Discussion

THP is a devastating injury with a high mortality rate, often exceeding 50%, due to severe hemorrhage, vascular disruption, and multi-organ involvement [3]. The patient in this case, a 29-year-old woman, sustained incomplete THP from a boating accident, resulting in massive soft tissue, osseous, and vascular injuries. Despite an ISS score of 45, her survival highlights the critical importance of prompt intervention, including rapid hemorrhage control, MTP, and a multidisciplinary approach involving trauma surgery, vascular surgery/interventional radiology, infectious disease, and orthopedics. Early recognition and management of the extensive vascular injuries, including internal iliac artery and vein ligation, were vital in stabilizing the patient hemodynamically.

The early removal of the limb due to the severity of the injuries aligns with literature suggesting that early amputation in cases of unsalvageable limbs can serve as a life-saving measure by preventing further hemorrhage and associated complications [2]. In this case, early limb removal not only controlled hemorrhage but also reduced the metabolic demands of the injured limb, mitigating the risk of SIRS and multiorgan failure [5]. By removing the nonviable extremity early, this patient avoided further deterioration, allowing a focus on stabilizing her overall condition and preventing life-threatening complications like coagulopathy and sepsis.

Flap necrosis is another major complication in the management of THP, with rates reported as high as 26% [11]. Preventing flap necrosis requires meticulous wound care and appropriate vascularization of the remaining tissue to ensure successful healing. In this case, comprehensive wound care operations including integration of advanced therapies like ECM beads, bilayer xenograft, and hyperbaric oxygen treatment and timely interventions helped mitigate the risk of flap necrosis, contributing to the patient’s positive outcome.

Infectious disease discussion

Preventing sepsis in a THP is challenging due to extensive soft tissue damage, open wounds, and specifically prolonged marine exposure in this patient, which heightened the risk of water-borne pathogens [9]. Reported rates of wound infection have been as high as 39% [11]. Marine environments can harbor organisms like *Vibrio *spp. and NTM that may precipitate necrotizing infections if not promptly managed. To mitigate these risks, broad-spectrum antibiotics with aquatic pathogen coverage were initiated, guided by cultures and sensitivities, alongside aggressive debridement of devitalized tissue.

The complexity of this case extended beyond superficial infections to deep intra-abdominal involvement. Repeated debridements revealed a persistently polymicrobial flora, including *E. gallinarum*, *S. maltophilia*, and various *Candida species*. Following the identification of *C. parapsilosis* and later *C. glabrata*, initial therapy with IV ampicillin, minocycline, and micafungin was initiated, with additional courses (ciprofloxacin and metronidazole) administered after abscess drainage. Although the STOP-IT trial supports shorter antibiotic courses, treatment duration was tailored to factors such as injury mechanism, wound contamination, prior exposures, and patient comorbidities [12]. Integration of operative findings, surgical consultation, imaging, and culture data with established guidelines to optimize source control guided specific pharmacotherapy and length of treatment. Notably, despite exposure to warm Florida waters, expected pathogens such as *Vibrio *spp. and *NTM* were absent, underscoring the variability in microbial colonization and the need for individualized antimicrobial strategies in complex trauma cases.

Retrospective considerations

In severe hemorrhagic shock within trauma settings, the use of resuscitative endovascular balloon occlusion of the aorta (REBOA) has become a common intervention. Although our facility does not currently utilize REBOA, it is important to consider its potential benefits and drawbacks. For instance, REBOA may offer an alternative to supraceliac compression during distal ligation; however, it is not without risks. Studies have indicated that approximately 12.1% of patients may experience vascular injuries, such as distal embolization and local vessel damage, which often require intervention [13]. Additionally, REBOA has been associated with limb compartment syndrome resulting from reperfusion injury following aortic occlusion [14].

The decision to perform a loop colostomy rather than an end colostomy was driven by the extensive pelvic destruction in this case, which would have made a reversal of an end colostomy considerably more challenging. Sphincteroplasty performed in this case also allowed for the potential of regaining sphincter function. The American Gastroenterological Association endorses loop colostomy for temporary diversions because this configuration facilitates a simpler and less invasive reversal process, reducing both operative time and hospital stay. Moreover, studies have shown that loop colostomies are associated with lower complication rates, such as reduced risks of suture line failure and fascial dehiscence, compared to end colostomies, which require more extensive surgery to re-anastomose the divided intestine [15,16].

In this case, flap amputation was decided over guillotine amputation due to several key considerations. The viability of the underlying muscles provided essential padding for the amputation site, and close monitoring of CPK levels allowed us to intervene early to prevent renal failure. Although guillotine amputation is advantageous in emergencies for rapid removal of necrotic or infected tissue [17], it generally requires additional procedures for definitive closure and is associated with a higher risk of infection and revision surgery [17,18]. In contrast, flap amputation has been shown to yield superior long-term functional outcomes, enhancing prosthetic use and limb function with comparable rates of surgical revision and limb salvage [19,20]. While complications such as flap necrosis and infection can occur with flap procedures, these are typically manageable, supporting its preference when immediate life-threatening conditions are not present [19-21].

Prehospital management of large soft tissue wounds benefits from dense packing with hemostatic dressings and the application of binders, both of which have been shown to enhance hemorrhage control [22,23]. Accordingly, we recommend that patients with such wounds be treated with these interventions during the prehospital period to optimize hemostasis.

## Conclusions

After extensive wound care and rehabilitation, including early physical therapy, occupational therapy, and coordination with an amputee mentor, the patient is now doing exceptionally well. Despite the severity of her injury and the subsequent surgeries, she has experienced minimal complications. Additionally, after anal manometry confirmed functional tone, she successfully underwent a loop colostomy reversal on November 11, 2024. She is now an active member of the trauma center as an amputee mentor less than one year after her injury. She is able to use a hemipelvis prosthetic with ambitions to be the world's first paralympian THP snowboarder.

This case illustrates the complexity of managing a THP. Rapid decision-making, a multidisciplinary approach, and early amputation in the setting of unsalvageable limbs can offer the best chance of survival. The patient's ongoing recovery, despite the severity of her injuries, demonstrates the importance of prompt intervention, comprehensive postoperative care, infection control strategies, and advanced wound management techniques.
